# Prevalence of overweight and obesity in Nigeria: Systematic review and meta-analysis of population-based studies

**DOI:** 10.1371/journal.pgph.0000515

**Published:** 2022-06-10

**Authors:** Innocent Ijezie Chukwuonye, Kenneth Arinze Ohagwu, Okechukwu Samuel Ogah, Collins John, Efosa Oviasu, Ernest Ndukaife Anyabolu, Ignatius Ugochukwu Ezeani, Gabriel Uche Paschal Iloh, Miracle Erinma Chukwuonye, Caleb Ogechi Raphael, Uwa Onwuchekwa, Umezurike Hughes Okafor, Clement Oladele, Emmanuel Chukwuebuka Obi, Chimezie Godswill Okwuonu, Okechukwu Iheji, Ogbonna Collins Nwabuko, Martin Anazodo Nnoli, Ikechi G. Okpechi

**Affiliations:** 1 Department of Internal Medicine, Federal Medical Centre Umuahia, Umuahia, Nigeria; 2 General Medicine and Emergency Care, West Cumberland Hospital, Cumbria, Emgland; 3 Department of Internal Medicine, University College Hospital, University of Ibadan, Ibadan, Nigeria; 4 Department of Paediatrics, Jos University Teaching Hospital Jos, Katon Rikkos, Nigeria; 5 Department of Internal Medicine, University of Benin Teaching Hospital, Benin, Nigeria; 6 Department of Internal Medicine, Chukwuemeka Odumegwu Ojukwu University Teaching Hospital Awka, Awka, Nigeria; 7 Department of Family Medicine, Federal Medical Centre Umuahia, Umuahia, Nigeria; 8 Department of Dietetics and Nutrition, Jos University Teaching Hospital, Jos, Nigeria; 9 Department of Internal Medicine, Abia State University Teaching Hospital, Aba, Nigeria; 10 Department of Internal Medicine, Enugu State University Teaching Hospital, Enugu, Nigeria; 11 Department of Internal Medicine, Federal Medical Centre Bida, Bida, Nigeria; 12 Department of Haematology, Federal Medical Centre Umuahia, Umuahia, Nigeria; 13 Department of Pathology, University of Calabar Teaching Hospital, Calabar, Nigeria; 14 Department of Medicine, University of Cape Town, Cape Town, South Africa; University of Washington, UNITED STATES

## Abstract

In Nigeria, several studies have assessed the prevalence of overweight/obesity with different reports. The purpose of this study was to use a systematic review and meta-analysis to analyze these overweight and obesity reports from different locations in Nigeria over the last ten years. In addition, there was a dearth of systematic reviews and meta-analyses on the prevalence, trends, and demographic characteristics of overweight and obesity in the country. This was a systematic review and meta-analysis of cross-sectional population-based studies among adult Nigerians on the prevalence of overweight/ obesity (defined by body mass index) published from January 2010 to December 2020. Relevant abstracts were scrutinized and articles that included adults of all age groups and were not restricted to a particular group of people (e.g. university community) were selected. Each article was scrutinized by more than 2 authors before selection. The prevalence of overweight/obesity among all participants, among men and among women in Nigeria and its 6 geopolitical zones was determined. All analyses were performed using STATA version 14 (Stata Corp. College Station, Texas, USA). Thirty-three studies were selected and the number of participants was 37,205. The estimated prevalence of overweight and obesity was 27.6%, and 14.5% respectively. The prevalence of overweight among men and among women was 26.3% and 28.3% respectively and, the prevalence of obesity among men and women was 10.9% and 23.0% respectively. The prevalence of overweight in the 6 geopolitical zones was Southeast 29.3%, Southwest 29.3%, South-south 27.9%, Northwest 27.2%, North-central 25.3%, Northeast 20.0% and obesity South-south 24.7%, Southeast 15.7%, Southwest 13.9%, Northwest 10.4%, North-central 10.2%, Northeast 6.4%. Egger’s tests showed no statistically significant publication bias among the studies that reported the overweight and obesity prevalence respectively (p = 0.225, P 0.350). The prevalence of overweight/obesity in Nigeria is high. The southern geopolitical zones had a higher prevalence of overweight/obesity.

## Introduction

The prevalence of overweight and obesity is on the increase worldwide, with serious public health implications. In the last three and half decades, the prevalence of obesity has increased steadily, with regard to the standard established by the World Health Organization (WHO) body mass index (BMI) categorization of obesity. The steady increase in the prevalence of overweight and obesity is global and the rate of increase in African countries like Nigeria is not lower than that observed in developed countries of the world [1,2]. In 2016, the WHO reported that about 1.9 billion adults were overweight (using BMI classification) and about a third of these (650 million) were obese globally. The prevalence of overweight was 38% (9% among men and 40% among women), while the prevalence of obesity was 13% (11% among men and15% among women) in adults aged 18 years and above in the WHO report [3,4].

The Global Burden of Disease Study in 2017 evaluated 84 risk factors and obesity was reported as one of five leading environmental, behavioral, and metabolic risks that drive injury and disease worldwide. Obesity was also observed to have the greatest relative increase in exposure since 1990 [5]. Obesity and being overweight are associated with a greater risk of non-communicable diseases such as cardiovascular diseases, diabetes mellitus, metabolic syndrome, chronic kidney disease, cancer, and musculoskeletal disorders. Cardiovascular disease was responsible for 41% of obesity-related deaths and 34% of obesity-related disability-adjusted life-years in obese people worldwide. In 2015, diabetes was the second largest cause of death from obesity-related causes^.^ [6]. In Nigeria, some of the co-morbidities reported included type 2 diabetes mellitus, hypertension, and dyslipidemia [7].

The theoretical framework for available multilevel factors driving adult obesity classifies the determinants of obesity into three levels: individual levels (genetic, ethnicity, socioeconomic, etc.), environmental factors, and lifestyle/behavioral/social factors. Changes in the risk factors at these different levels in the system affect the development of obesity in individuals [8].

In Nigeria, some risk factors for obesity have been reported and these; include gender, age, locality (urban community), decreased physical activity, educational status, high income, and diet [9–12]. Increased dietary consumption of energy-dense foods, high levels of refined sugar and saturated fats (fast food) and sedentary lifestyles are recognized as some of the major causes of the increased prevalence of obesity in Nigeria [10]. There has also been a rapid increase in the number of eateries that sell fast food in most urban communities in the country within the last three decades with associated increased patronage by the upper and middle class that can afford it. A study in Nigeria reported that the prevalence of obesity in low, middle, and upper-income classes were 12.2%, 16%, and 20%, respectively [13], indicating that the prevalence was higher in the upper and middle class in the country.

Nigeria has strategic direction documents on promoting physical activities, nutritional counseling, adhering to dietary guidelines, and implementing mandatory nutritional labeling. All these are captured in the country’s health and nutritional policies. The problem however is that more attention is currently being paid to undernutrition [14]. In order to convince policy-makers to pay more attention to overweight and obesity reliable statistics highlighting obesity as a serious public health problem in Nigeria are needed. The goal of this study was to assess the prevalence of overweight and obesity in Nigeria and its six geopolitical zones using data from multiple population-based studies conducted across the country. In addition, we also intended to test the hypothesis that the prevalence of obesity had increased in the last decade when compared to preceding decades. A recent reliable estimate of the prevalence of overweight and obesity among the adult population in the country will contribute to the statistics needed to sway policymakers in the country to take urgent and substantial action on the increasing prevalence of obesity.

## Methodology

This was a systematic review and meta-analysis study and the guidelines of the Preferred Reporting Items for Systematic Reviews and Meta-Analysis (PRISMA) [15] were adopted and the PRISMA checklist adhered to http://www.prisma-statement.org/. The study focused on overweight and obesity defined by BMI and BMI was classified as follows: overweight, BMI of 25–29.9 kg/m^2^; and obesity, BMI of 30 kg/m ^2^ and above [16].

### Literature review

A literature search for population-based studies on overweight and obesity, published from January 1, 2010, to December 31st, 2020 on adult Nigerians using the search terms, “obesity” “body mass index” and “overweight”. These search terms were used in Google Scholar, PubMed, and Embase search engines to retrieve all potentially relevant English articles. The key search words were combined with Nigeria. Bibliographies of some of the authors were also searched and retrieved abstracts were also scrutinized. In order to eliminate difficulties in analyzing the data, we only paid attention to surveys that made use of BMI in the definition of overweight and obesity, or where both BMI and another method were used only the BMI results were extracted. Waist circumference is another common method of assessing obesity in Nigeria. However, in most studies that were available on abdominal obesity; the protocols for measuring waist circumference were not the same, making it difficult to compare most of the studies. In addition, there is no universally acceptable cut-off criterion for defining abdominal obesity for men and women, due to the existence of different criteria (e.g. The Adult Treatment Panel III and the International Diabetes Foundation) [17]. This is why we focused only on studies that used BMI in defining overweight and obesity.

### The criteria for the inclusion of articles

1. The study must be a population-based study with results on the prevalence of overweight.
2. The location of the study in Nigeria must be stated.
3. The period of publication of the study must be within the range of January 1st, 2010 –December 31st, 2020.
4. The study must include adults (18 years of age and older) men and women and people of all ages
5. BMI should be used to stratify overweight and obesity.
6. The study should not be limited to a particular group of people (e.g. factory workers, the university community, or the market community).

### Validation of search results

Search results were validated in three stages, as follows:

1. The abstracts of articles on population-based studies on adults found with the above search terms and published between January 1, 2010, and December 31, 2020, were read and those that met the criteria were selected.
2. Abstracts that did not meet the inclusion criteria were discarded.
3. Obtained full-text studies that met the inclusion criteria were reviewed by at least three authors independently and any knotty issue surrounding any article under consideration was discussed by the authors and the final decision on the article was taken by consensus.
4. A PRISMA flow diagram of steps taken in arriving at the number of included studies was drawn at the end of the screening.

### Data extraction

Data extracted from the studies that met the selection criteria included the study community, the geopolitical zone of Nigeria where the study was carried out, year of publication of the article, study design, sample size, mean age (years) of participants in the study, and the prevalence of overweight and obesity among adults in the study based on BMI only.

### Statistical analysis

The standard error (SE) and effect size (ES) of the prevalence estimates were calculated using metaprop_one which is the update command of metaprop for performing a meta-analysis of proportions. Heterogeneity chi-square χ^2^ test and τ^2^ Tau^2^ statistic (τ^2^) was used to assess heterogeneity and the estimate of between-study variance. P-values of less than 0.05 were considered as heterogeneity. The *I*^*2*^ statistic was also done for each of the pooled estimates to test for variation in ES attributable to heterogeneity. As the differences between the studies were very large (92–98% inconsistency), a random-effects model was used to pool the prevalence of overweight and obesity in Nigeria. Metareg, which performs random-effects meta-regression, was done to assess heterogeneity and combinability. Freeman-Tukey transformations were done to stabilize the geopolitical variances to arrive at the overall prevalence of overweight and obesity in Nigeria. Geopolitical zone-wise pooled estimates weighted by population size in each study place within a given zone (Southeast; South-south, South-west; North-central, Northeast and North-west) for the prevalence of overweight and obesity were also calculated. All analyses were done using STATA version 14 (Stata Corp. College Station, Texas, USA).

### Publication bias

Inspection of the funnel plot and Egger’s bias test were used to assess potential bias in the study [18].

## Results

### Study selection

**Fig 1:**
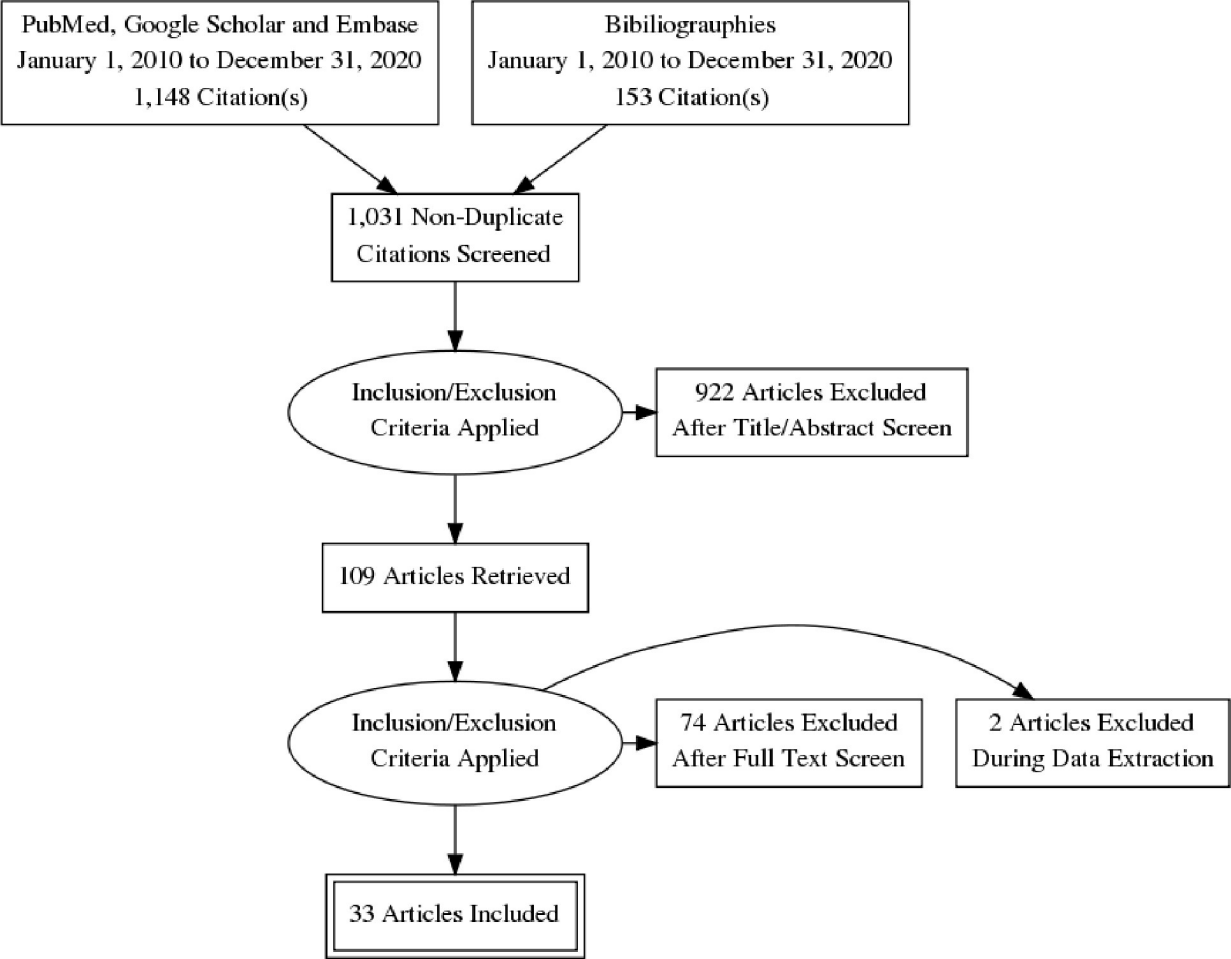
PRISMA flow diagram. Graphical representation of the flow of citations reviewed in the course of the systematic review and meta-analysis. Abbreviation: Preferred items for systematic review and meta-analysis (PRISMA).

The number of abstracts on population-based studies on overweight and obesity identified in the study from the databases was 1,148 and 153 articles were identified from bibliographies. A total of 922 abstracts were excluded from 1,031 non-duplicate abstracts and a total of 109 original articles were retrieved. Thirty of the 109 retrieved articles were from the Southeast (SE), 17 from the South-south (SS), 33 from the Southwest (SW), 13 from the North-central (NC), 6 from the Northeast (NE), and 10 from the Northwest (NW) geopolitical zone. Thirty-three articles that fulfilled all the inclusion criteria were finally selected (PRIMA diagram Fig 1).

### Study characteristics

The total number of the participants that took part in the 33 included articles was 37,205 (SE = 17,422, SS = 5,313, SW = 8,488, NC = 1,414, NE = 2,822, NW 1,566) The 33 studies were population-based observational cross-sectional designs. They were all community-based studies; however, 2 of the studies were state-wide studies [10,11] and there was no geopolitical zone, regional or nationwide study. Ten of the articles [9–11,19–25] (30.30%) were from the SE, 5 (15.15%) were from the SS [12,26–29], 9 (27.27%) were from the SW [30–38], 3 (9.09%) were from the NC [39–41], 2 (6.06%) were from the NE [42,43]and 4 (12.12%) from the NW geopolitical zone [44–47]. Five of the 33 studies [19,27,29,32,34] did not report the prevalence of overweight among the participants. The prevalence of overweight and obesity among the men and women genders with the sample sizes of the men and women that took part in the study was reported by 10 and 15 studies, respectively (Table 1).

**Table 1:** The prevalence of overweight and obesity from population based studies in Nigeria.

Zones	Ref.	Author (year)	Sample size	Study design	Mean age(years)	Obesity prevalence (%)	Overweight prevalence (%)
South East	9	Ijoma et al. (2019)	605	Cross sectional	44.5	19.5(M = 7.9,F = 24.9)	29.4(M = 28.3,F = 30.0)
	10	Chukwuonye et al. (2015)	2928	Cross sectional	41.7 ± 18.5	12 b.3(M = 7.8,F = 16.4)	28.2(M = 28.8,F = 27.7)
	11	Chigbu et al. (2018)	6628	Cross sectional		6.8	19.0
	19	Gladys et al. (2011)	218	Cross sectional		13.3(M = 10.1,F = 14.8)	
	20	Ezeala-Adikaibe et al. (2016)	774	Cross sectional	43.9	17.8(M = 7.2,F = 23.7)	27.9(M = 25.5,F = 29.2)
	21	Fatai and Udoji (2015)	1521	Cross sectional	43.98	26.9(M = 19.6,F = 36.0	31.2(M = 32.3,F = 29.8)
	22	Ijoma et al. (2020)	210	Cross sectional	51.24 ± 16.24	10.9(M = 10.9,F = 10.9)	28.0(M = 27,F = 28)
	23	Okafor et al. (2011)	898	Cross sectional	48.7 ± 12.9	21.2	37.8
	24	Ulasi et al. (2010)	1458	Cross sectional	43.8 ± 13.7	17.3	31.6
	25	Ulasi et al. (2013)	2182	Cross sectional	43.7 ± 13.2	14.9	31.9
South-south	12	Adienbo et al. (2012)	304	Cross sectional	37.66 ± 14.94	49.34(M = 35.51,F = 64.49)	22.4
	26	Nwafor et al. (2015)	250	Cross sectional		16.4(M = 5.6,F = 10.8)	41.2(M = 15.2,F = 26.0)
	27	Egbe et al. (2014)	1134	Cross sectional		27.4(M = 22.3,F = 34.2)	
	28	Isara et al. (2015)	845	Cross sectional	56.4 ± 16.3	10.6	21.8
	29	Ekpenyong et al. (2012)	2780	Cross sectional		25.00	
South-west	30	Chinedu et al. (2013)	489	Cross sectional		18.0	31.0
	31	Raimi and Dada (2018)	552	Cross sectional	39.9 ±15.5	18.3(M = 8.8,F = 27.7)	34.8(M = 30.9,F = 37.6)
	32	Oluyombo et al. (2015)	750	Cross sectional	61.7 ± 18.2	8.5	
	33	Abiodun et al. (2014)	776	Cross sectional	42.6 ± 14.3	17.5	29.9
	34	Oluyombo et al. (2016)	1083	Cross sectional	55.1 ± 19.9	5.7	
	35	Amira et al. (2012)	1368	Cross sectional	41.9 ± 12.9	22.2(M = 15.7,F = 29.5)	32.7(M = 33.3,F = 31.9)
	36	Akinwale et al. (2013)	2434	Cross sectional		19.1	36.2
	37	Adebayo et al. (2014)	777	Cross sectional	36.3 ± 14.3	8.4(M = 10.3,F = 6.9)	20.8(M = 22.3,F = 19.0)
	38	Asekun-Olarinmoye et al. (2013)	259	Cross sectional	49.7 ± 1.6	11.5	19.6
North central	39	Etukumana et al	750	Cross sectional study	39.42±16.17	8 (M = 3.2, F 13.1)	23.3
	40	Sola et al. (2011)	435	Cross sectional	24.2 ± 0.2	4.0	22.0
	41	Adediran et al. (2012)	229	Cross sectional		22.3(M = 8,F = 36.2)	32.3
North-east	42	Oyeyemi et al. (2012)	1818	Cross sectional	32.3 ± 10.0	8.1	22.8
	43	Adedoyin et al. (2012)	1004	Cross sectional	41.5 ± 13.5	3.8	15.4
North-west	44	Wahab et al. (2011)	300	Cross sectional	37.6 ± 10.6	21.0(M = 9.3,F = 29.8)	53.3(M = 41.9,F = 62.0)
	45	Dahiru and Ejembi, (2013)	199	Cross sectional	39.9 ± 15.6	7.0	26.9
	46	Makusidi et al. (2013)	535	Cross sectional	37.0 ± 17.0	6.7	12.3
	47	Ramalan et al. (2019)	532	Cross sectional	38.9 ± 15.9	9. 2(M = 4.3,F = 13.6)	20.9(M = 15.4,F = 24.4)

M = Male, F = Female.

Two articles were excluded during the data extraction. One of the articles [48] was from the same community-based study from which an article had been selected [10]. The second study included pregnant women in the study and also excluded people living with diabetes mellitus and other chronic diseases [49].

### The prevalence of overweight in Nigeria

**Fig 2:**
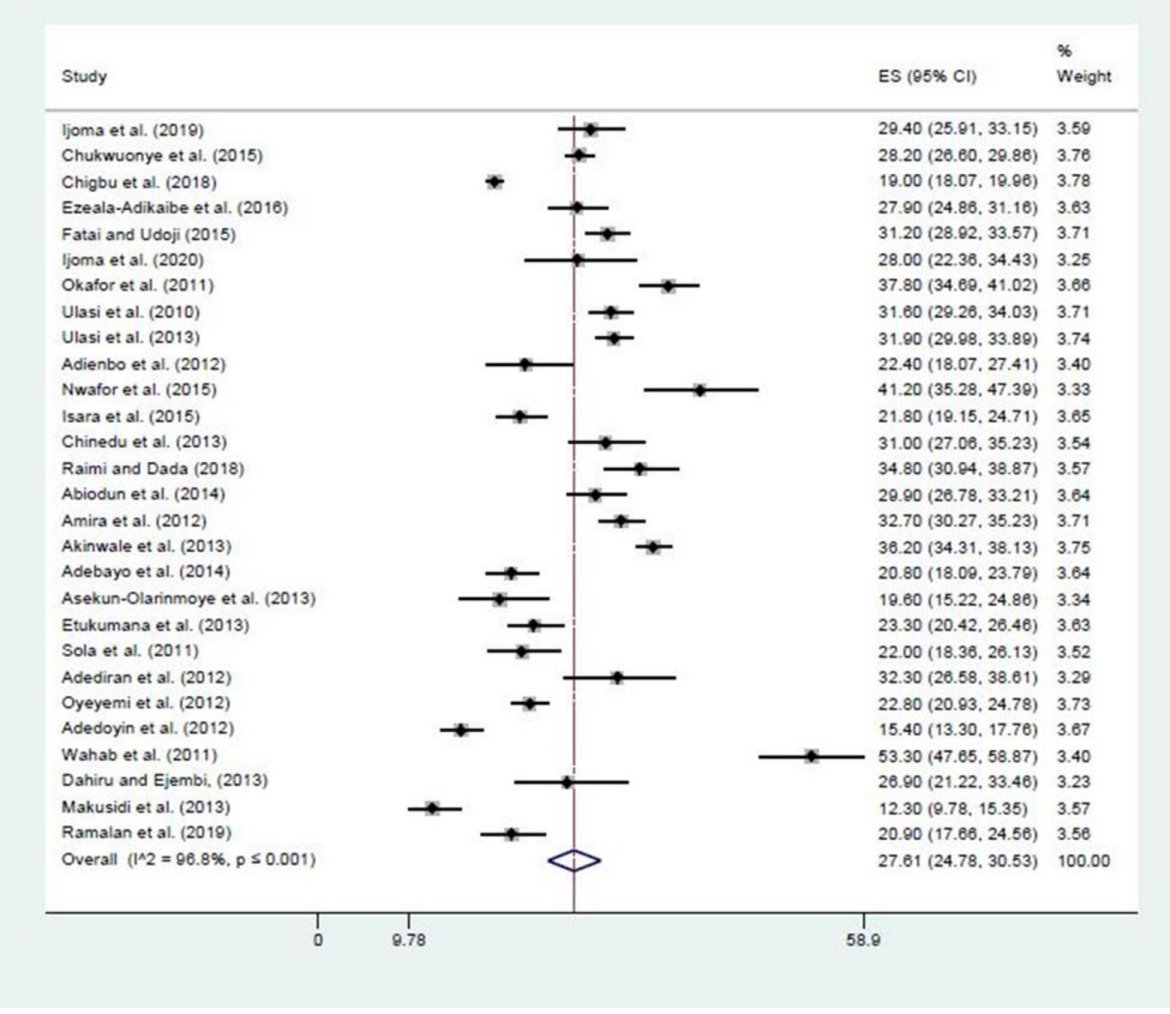
The prevalence of overweight among adults in Nigerians.

**Fig 3:**
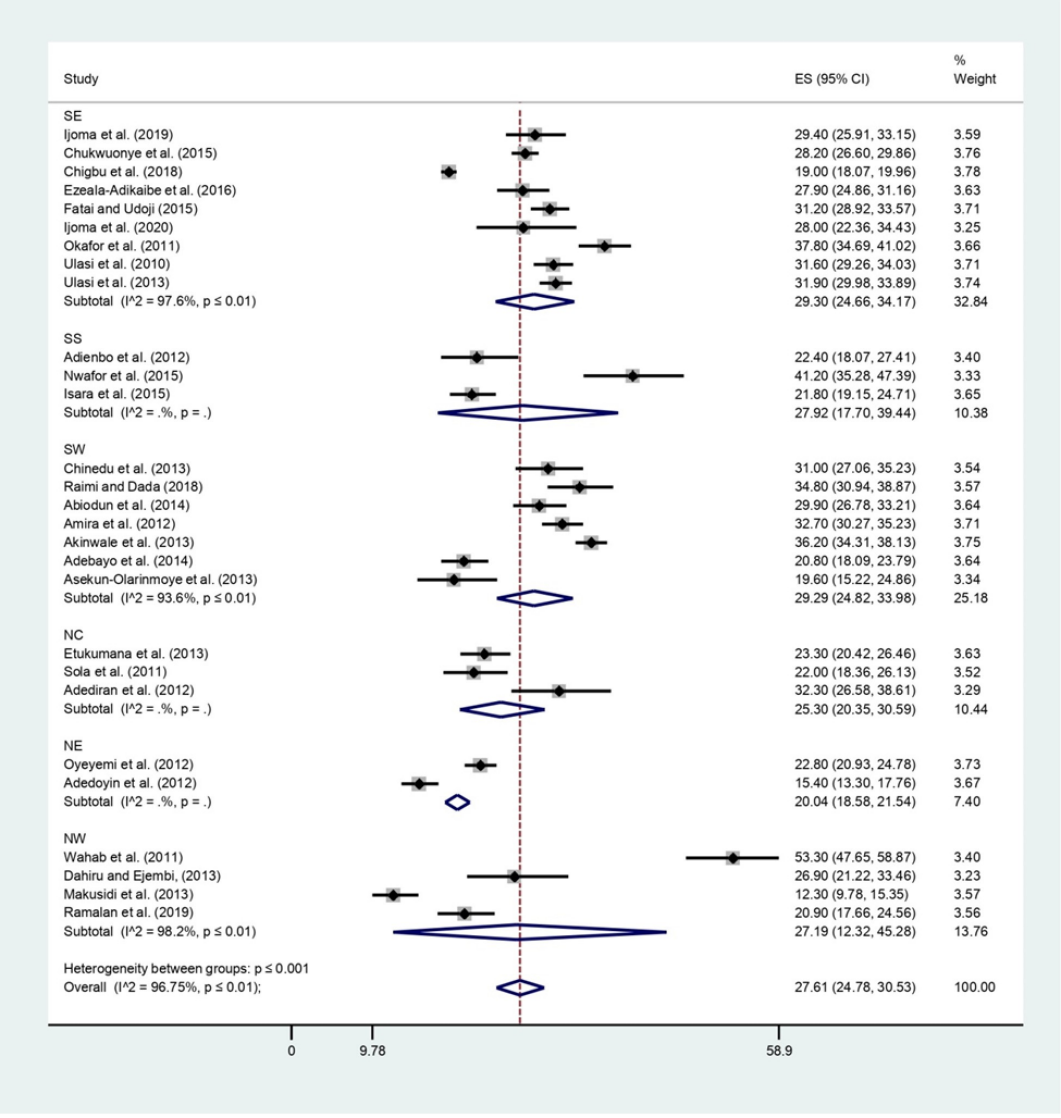
The prevalence of overweight in the six geopolitical zones in Nigeria.

The prevalence of overweight from the studies ranged from 12.3% in NW 46 to 41.2% in SS26 geopolitical zones. Heterogeneity was significantly present among the geo-political zones. After stabilizing the regional data using Freeman-Tukey transformations, the overall prevalence of overweight found in Nigeria from this study was 27.6% (95% CI: 24.8–30.5; I2 = 96.75, P <0.001). The prevalence of overweight people was highest in SE and SW, with both having 29.3% [95% CI: (24.7–34.2) and (24.8–34.0) respectively]. While NE had the lowest prevalence 20.0% (95% CI: 18.6–21.5) (See Table 2, Figs 2 and 3 for more details).

**Table 2:** Pooled estimates of the prevalence of overweight in Nigeria.

	Prevalence (%)	(95% CI)	I^2^%	p-value	Cases
**Nationwide**					
Overweight	27.6	(24.8–30.5)	96.75	0.00	8,300
**Geopolitical zone**					
North-central	25.3	(20.4–30.6)	-	-	344
North-east	20.0	(18.6–21.5)	-	-	569
North-west	27.2	(12.3–45.3)	98.2	0.00	390
South-east	29.3	(24.7–34.2)	97.6	0.00	4,508
South-west	29.3	(24.8–34.0)	93.6	0.00	2,117
South-south	27.9	(17.7–39.4)	-	-	355

### The prevalence of obesity in Nigeria

**Fig 4:**
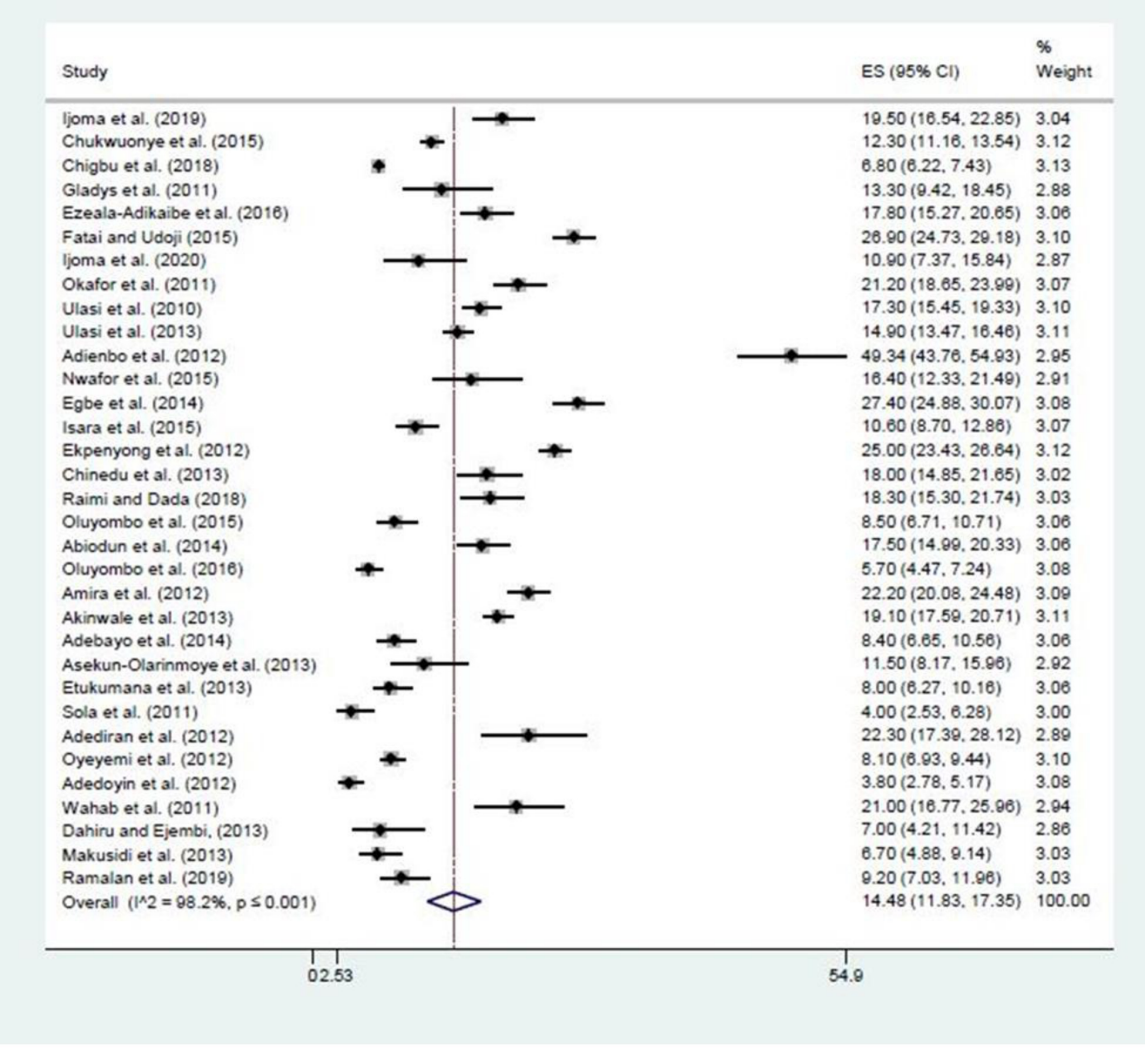
The prevalence of obesity among adults in Nigeria.

**Fig 5:**
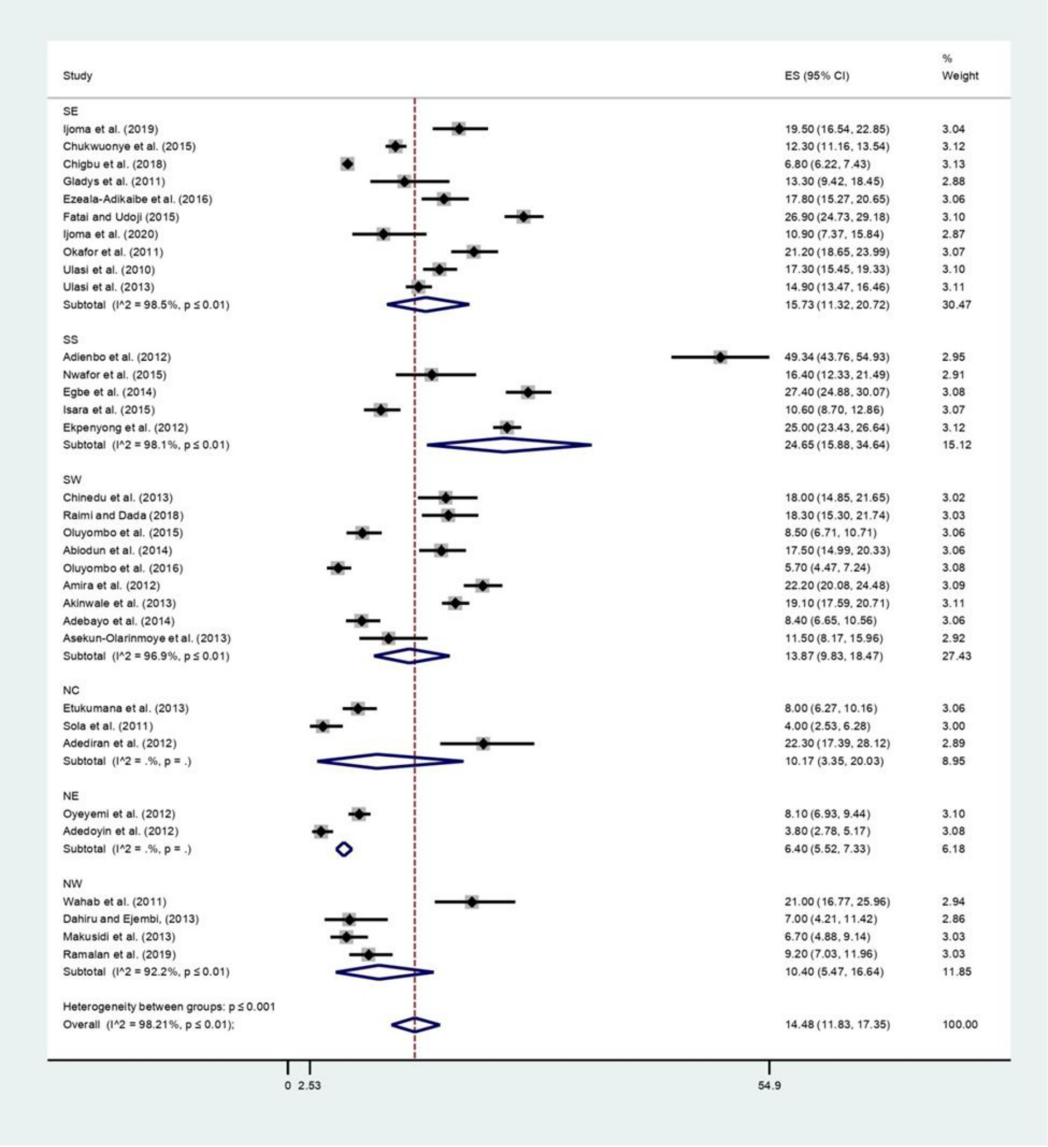
The prevalence of obesity among adults in the six geopolitical zones of Nigeria.

The prevalence of obesity from the studies ranged between 4.0% in NC 38 to 49.3% in SS9 (The overall pooled crude prevalence of obesity in Nigeria was 14.5% (95% CI: 11.8–17.4; I2 = 98.2%, P<0.001). There was a significant difference in the pooled prevalence across the geopolitical zones. SS zone had the highest prevalence of 24.7% (95% CI: 15.9–34.6; I2 = 98.1%, P<0.001). NE had the lowest prevalence of obese people at 6.4% (95% CI: 5.5–7.3; I2 = 0%). (See Table 3, Figs 4 and 5 for more details).

**Table 3:** Pooled estimates of the prevalence of obesity in Nigeria.

	Prevalence (%)	(95% CI)	I^2^%	p-value	Cases
**Nationwide**					
Obesity	14.5	(11.8–17.4)	98.2	0.00	5,378
**Geopolitical zone**					
North-central	10.2	(3.4–20.0)	-	-	128
North-east	6.4	(5.5–7.3)	-	-	185
North-west	10.4	(5.5–16.6)	92.2	0.00	162
South-east	15.7	(11.3–20.7)	98.5	0.00	2,302
South-west	13.9	(9.8–18.5)	96.9	0.00	1,314
South-south	24.7	(15.9–34.6)	98.1	0.00	1,286

### Publication bias

**Fig 6:**
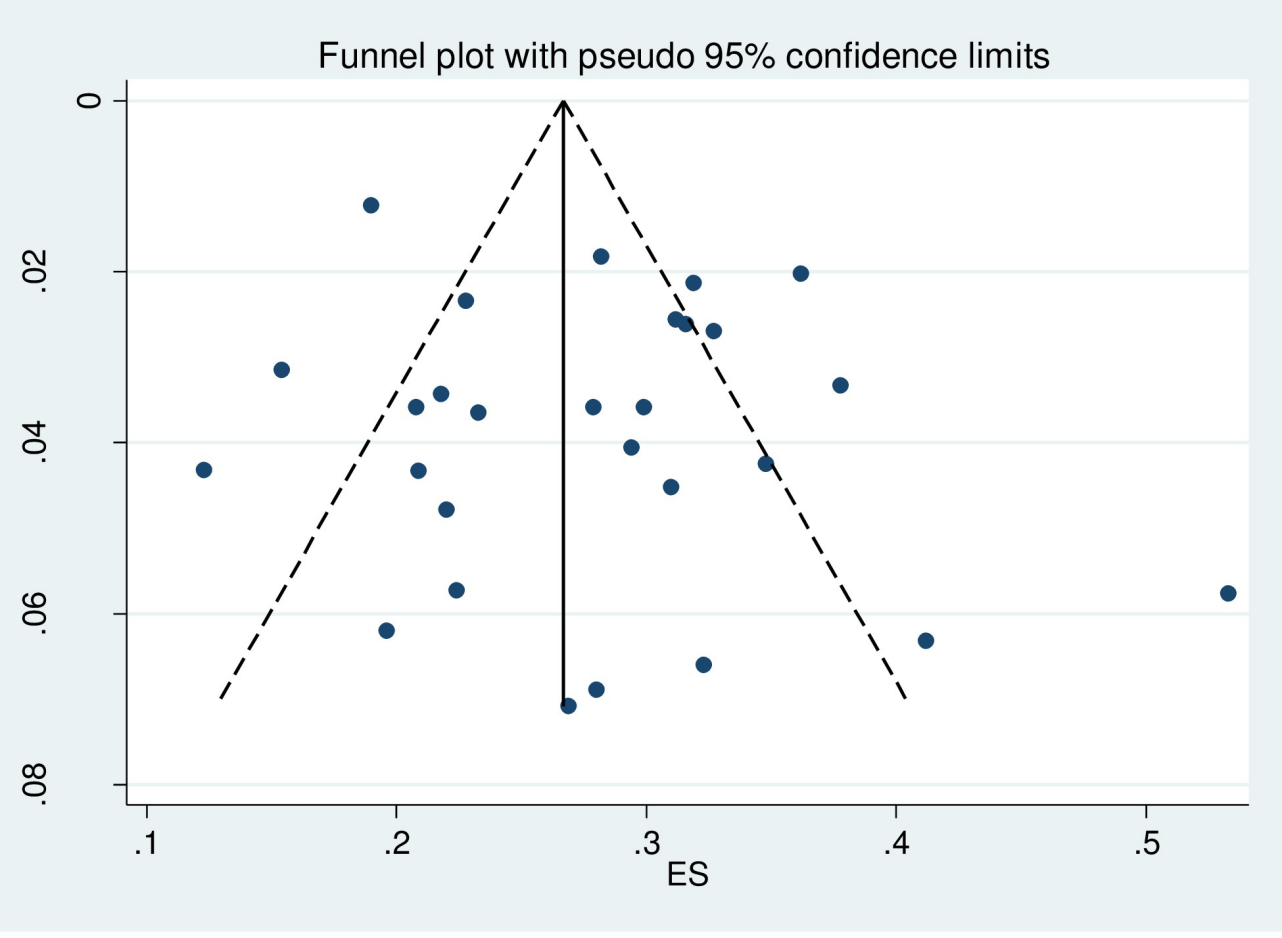
Distribution of studies included in the prevalence of overweight among adults in Nigeria in the meta funnel plot.

**Fig 7:**
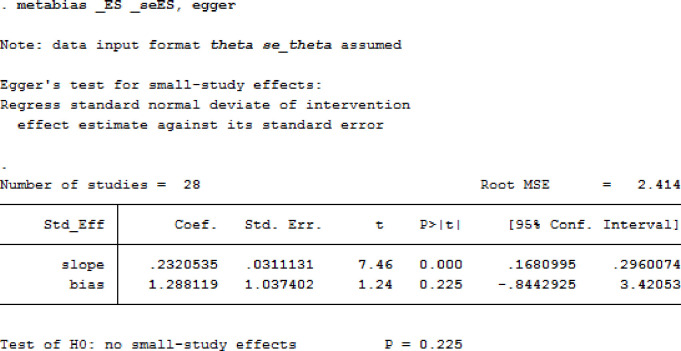
Egger’s test for detection of publication bias for studies included in the prevalence of overweight among adults in Nigeria.

**Fig 8:**
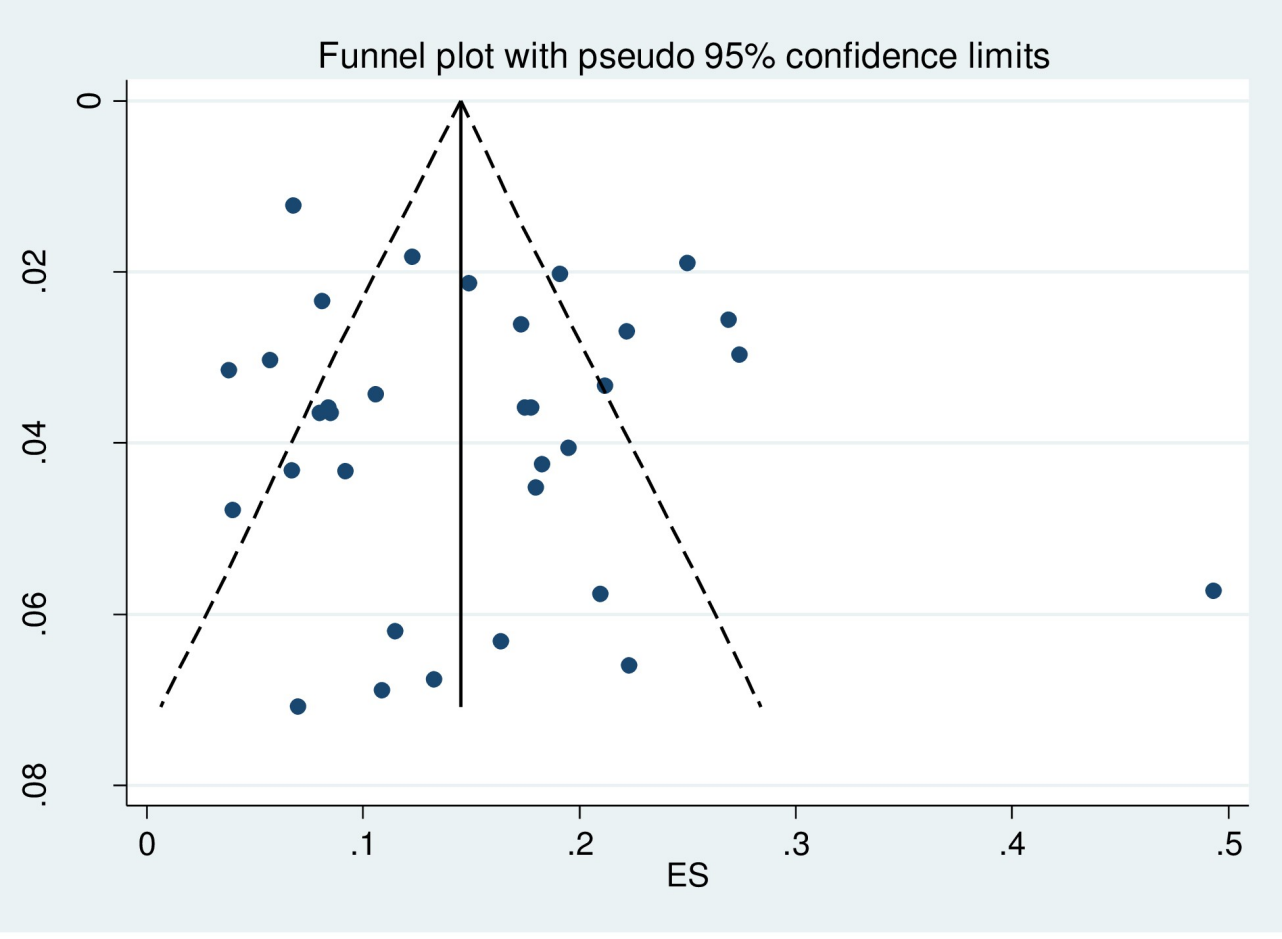
Distribution of studies included in the prevalence of obesity among adults in Nigeria in the meta funnel plot.

**Fig 9:**
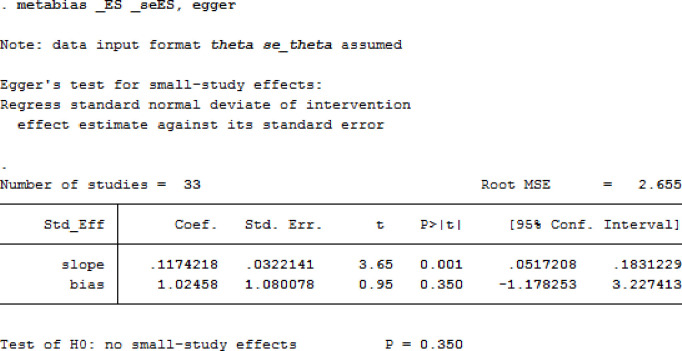
Egger’s test for detection of publication bias for studies included in the prevalence of obesity among adults in Nigeria.

The publication bias among studies included for overweight and obesity was determined using the funnel plot and Egger’s tests. The results of Egger’s tests for the funnel plot showed that there was no statistically significant publication bias in the studies that reported the overweight and obesity prevalence, respectively (p = 0.225 for overweight and p = 0.350 for obesity). (See Figs 6 and 7 for overweight people and Figs 8 and 9 for obese people).

### The prevalence of overweight among men and among women in Nigeria

The pooled prevalence of overweight among men and women was determined by 10 studies that met the inclusion criteria. The prevalence of overweight among men was 26.3% (95% CI: 22.9–29.9; I2 = 82.83%, P<0.001) in Nigeria. Among women the prevalence was 28.3% (95% CI: 25.6–31.2; I2 = 77.61%, P<0.001). Geopolitical zone-wise, SS had the lowest prevalence of overweight among men at 15.2% (95% CI: 9.1–24.3), while the South-east region had the highest at 29.2% (95% CI: 26.9–31.6; I2 = 31.33%, P>0.05). Among women, the prevalence of overweight was 24.4% (20.0–29.4) in the NW geopolitical zone as the lowest and 29.2% (95% CI: 19.4–40.0) in the SW as the highest. (See Tables 4 and S1 and S1 and S2 Figs).

**Table 4:** Pooled estimates of the prevalence of overweight among men and women in Nigeria.

	Male		Female	
	Prevalence % (95% CI)	I^2^%, p-value	Prevalence % (95% CI)	I^2^%, p-value
**Nationwide** overweight	26.3 (22.9–29.9)	82.83, 0.00	28.3 (25.6–31.2)	77.61, 0.00
**Geopolitical zone**				
North-central	-	-	-	-
North-east	-	-	-	-
North-west	15.40 (11.1–20.9)	-	24.4 (20.0–29.4)	-
South-east	29.2 (26.9–31.6)	31.33, 0.21	28.6 (27.1–30.2)	0.00, 0.81
South-west	28.8 (21.9–36.1)	-	29.2 (19.4–40.0)	-
South-south	15.2 (9.1–24.3)	-	26.0 (20.0–33.2)	-

### The prevalence of obesity among men and among women in Nigeria

The pooled prevalence of obesity among men and among women was determined by 15 studies. A higher pooled prevalence of obesity was observed among women 23.0% (95% CI: 17.2–29.4; I2 = 97.0%, P<0.001), compared to the men 10.9% (95% CI: 17.2–29.4; I2 = 94.2%, P<0.001) in Nigeria. Regarding the different geo-political zones the rates of obesity prevalence were 13.6% (95% CI: 10.3–17.8) in the NW as the lowest and 34.8% (95% CI: 11.3–63.3) in the SS as the highest. Among the men the lowest prevalence was 4.0% (95% CI: 2.4–6.0) in the NC while the highest was 19.8% (95% CI: 8.0–35.1) in the SS. (See Tables 5 and S2 and S3 and S4 Figs).

**Table 5:** Pooled estimates of the prevalence of obesity among men and women in Nigeria.

	Male		Female	
	Prevalence % (95% CI)	I^2^%, p-value	Prevalence % (95% CI)	I^2^%, p-value
**Nationwide** obesity	10.9 (17.2–29.4)	94.2, 0.00	23.0 (17.2–29.4)	97.0, 0.00
**Geopolitical zone**				
North-central	4.0(2.4–6.0)	-	17.8 (14.5–21.4)	-
North-east	-	-	-	-
North-west	4.3 (7.6–14.8)	-	13.6 (10.3–17.8)	-
South-east	10.3 (5.7–15.9)	93.1, 0.00	20.8 (14.1–28.4)	95.7, 0.00
South-west	11.7 (7.8–16.3)	-	20.1 (7.1–37.5)	-
South-south	19.8 (8.0–35.1)	-	34.8 (11.3–63.3)	-

## Discussion

This systematic review and meta-analysis highlighted the prevalence of overweight and obesity among adults in Nigeria and its 6 geopolitical zones based on published studies from January 1, 2010, to December 31, 2020. It is the first systematic review and meta-analysis in the country that delved into the prevalence of overweight and obesity among adults in each of the 6 geopolitical zones in Nigeria from the literature search. The total number of participants that took part in this study was 37,205 and the estimated pooled prevalence of overweight among adults in Nigeria ranged from 12.3% to 41.2% and the prevalence of obesity among adults in Nigeria ranged from 4.0% to 49.3%. In an earlier systematic review [17], the prevalence of overweight among adults in Nigeria ranged from 20.3%–35.1%, while the prevalence of obesity ranged from 8.1%–22.2%. The observed differences between both studies might be due to the reported rising level of overweight and obesity [3] and also because this study captured more recent studies and covered a wider period.

The estimated pooled prevalence of overweight and obesity among adults in Nigeria in this study was 27.6%, and 14.5% respectively. There is a dearth of systematic reviews and meta-analyses on the prevalence of overweight and obesity in Nigeria. In an earlier meta-analysis study by Abubakari et al, [50] the prevalence of obesity among Nigerian adults was 8.8% (CI 7.0–10.6) in 2000, and obesity in Ghanaian adults (> or = 25 years) was 14.1% (CI 13.1–15.1%) in 1998, A comparison of the reports showed that the prevalence of obesity had risen in Nigeria. A meta-analysis in Ghana by Ofori-arenso et al [51] reported a prevalence of overweight and obesity among adults in Ghana of 25.4% and 17.1%, respectively. The observed results were close to those observed in this study. The prevalence of overweight and obesity in Nigeria and Ghana were similar which suggested this might be the pattern in the West African region. In a meta-analysis by Kassie et al [4] in Ethiopia, involving published studies on the prevalence of overweight and obesity among adults in Ethiopia covering almost the same period as our study (from January 2010 –March 2020) the estimated pooled prevalence rate of overweight and obesity was 19% and 5.4% respectively. The observed results were much lower than those observed in Nigeria and Ghana (West African countries) and tend to suggest that the prevalence of overweight and obesity among adults in West African countries might be markedly higher than that obtainable in countries in the Horn of Africa. Ofori-arenso et al [51] and Kassie et al [4] also reported an increased prevalence of overweight and obesity in Ghana and Ethiopia respectively. These findings strongly suggest that there is a rapid rise in the prevalence of overweight and obesity in most or all African countries primarily due to lifestyle modifications and other factors. The prevalence rate of overweight and obesity observed among adults in Nigeria in this study was not lower than that reported by the WHO [3] in 2016. This was a pointer that the prevalence of overweight and obesity in Nigeria and some other African countries like Ghana was on par with the WHO [3] reports.

The prevalence of overweight among men and among women was 26.3% and 28.3% respectively. In addition, the prevalence of obesity among men and women was 10.9% and 23.0% respectively. These results showed that the prevalence of overweight and obesity was higher among women. These findings were in keeping with those observed by the WHO [3] and other studies [4,10] from Africa. Magemba et al [52] reported that the use of hormonal contraceptives and marriage were among the risk factors for overweight and obesity among women in Zimbabwe. However, more research is needed to determine the reasons for the difference in the prevalence of obesity between men and women in African countries. This is the first study from the literature search that is reporting the prevalence of overweight/obesity among men and women in Nigeria and there was none to compare our results with from the literature search.

Heterogeneity was observed in the prevalence of overweight/obesity among adults in the 6 geopolitical zones in Nigeria. The prevalence of overweight was (SE 29.3%, SW 29.3%, SS 27.9%, NW 27.2%, NC 25.3%, NE 20.0%) and obesity (SS 24.7%, SE 15.7%, SW 13.9%, NW 10.4%, NC 10.2%, NE 6.4%). In both overweight and obesity, the differences between the regions were statistically significant (p <0.05). The southern geopolitical zones of the country had higher prevalence rates of overweight/obesity when compared to the northern geopolitical zones. However, there was no previous study to compare the results from the literature search. The reasons for the higher prevalence of overweight and obesity in the southern geopolitical zones were multifactorial, and these included higher patronage of fast food in the southern geopolitical zone, increased sedentary lifestyle due to more affluence and industrialization. In addition, differences in dietary habits and a higher level of education in the southern region may also be part of the risk factors.

## Conclusion

The prevalence of overweight and obesity in Nigeria was high and had increased over the decades. There is a need to stem the trend because the cost implications are huge. The cost implications of overweight and obesity can be classified as direct or indirect costs. The costs of preventive, diagnostic, and treatment services constitute the direct cost and the cost of morbidity and mortality constitute the indirect cost. Morbidity costs are defined as the income lost from decreased productivity, restricted activity, absenteeism, and hospital admission days. The value of future income lost by the premature death of obese patients is known as mortality costs [17]. In the United States, obesity-related medical care costs in 2008, were estimated to be $147 billion and the annual nationwide productivity costs of obesity-related absenteeism ranged between $3.38 billion ($79 per obese individual) and $6.38 billion ($132 per obese individual) [53]. The direct and indirect cost of obesity in Nigeria is not known but is expected to be huge considering the high prevalence of obesity in Nigeria and also the fact that Nigeria is the most populous black nation on Earth. Obesity’s increased prevalence in Nigeria, however, is matched by rising levels of obesity’s co-morbidities, such as hypertension and diabetes mellitus [54,55]. There is a need for the various levels of governments and other key stakeholders in Nigeria and other African countries to invest more in preventive, diagnostic, and treatment of obesity and its comorbidities.

### Limitations

Five out of the 33 selected studies did not report the prevalence of overweight among the study participants. In addition, only 10 and 15 studies adequately reported the prevalence of overweight and obesity, respectively, among men and women in their various study populations.

### Recommendation

Based on the high and rising levels of overweight and obesity observed in this study, we urge that policymakers in Nigeria and other sub-Saharan African countries pay more attention to overweight and obesity due to the fact that they pose serious public health problems.

## Supporting information

S1 Fig The prevalence of overweight among men in Nigeria. (TIF)

S2 Fig The prevalence of overweight among women in Nigeria. (TIF)

S3 Fig The prevalence of obesity among men in Nigeria. (TIF)

S4 Fig The prevalence of obesity among women in Nigeria. (TIF)

S1 Table The prevalence of overweight among men and women in population based studies in Nigeria. (DOCX)

S2 Table The prevalence of obesity among men and women in population based studies in Nigeria. (DOCX)
