# Supplementary figures and images for: Prevalence of overweight and obesity in Nigeria: Systematic review and meta-analysis of population-based studies
Source: PLOS Glob Public Health. 2022 Jun 10;2(6):e0000515. doi: 10.1371/journal.pgph.0000515 (PMC10021772; doi:10.1371/journal.pgph.0000515)

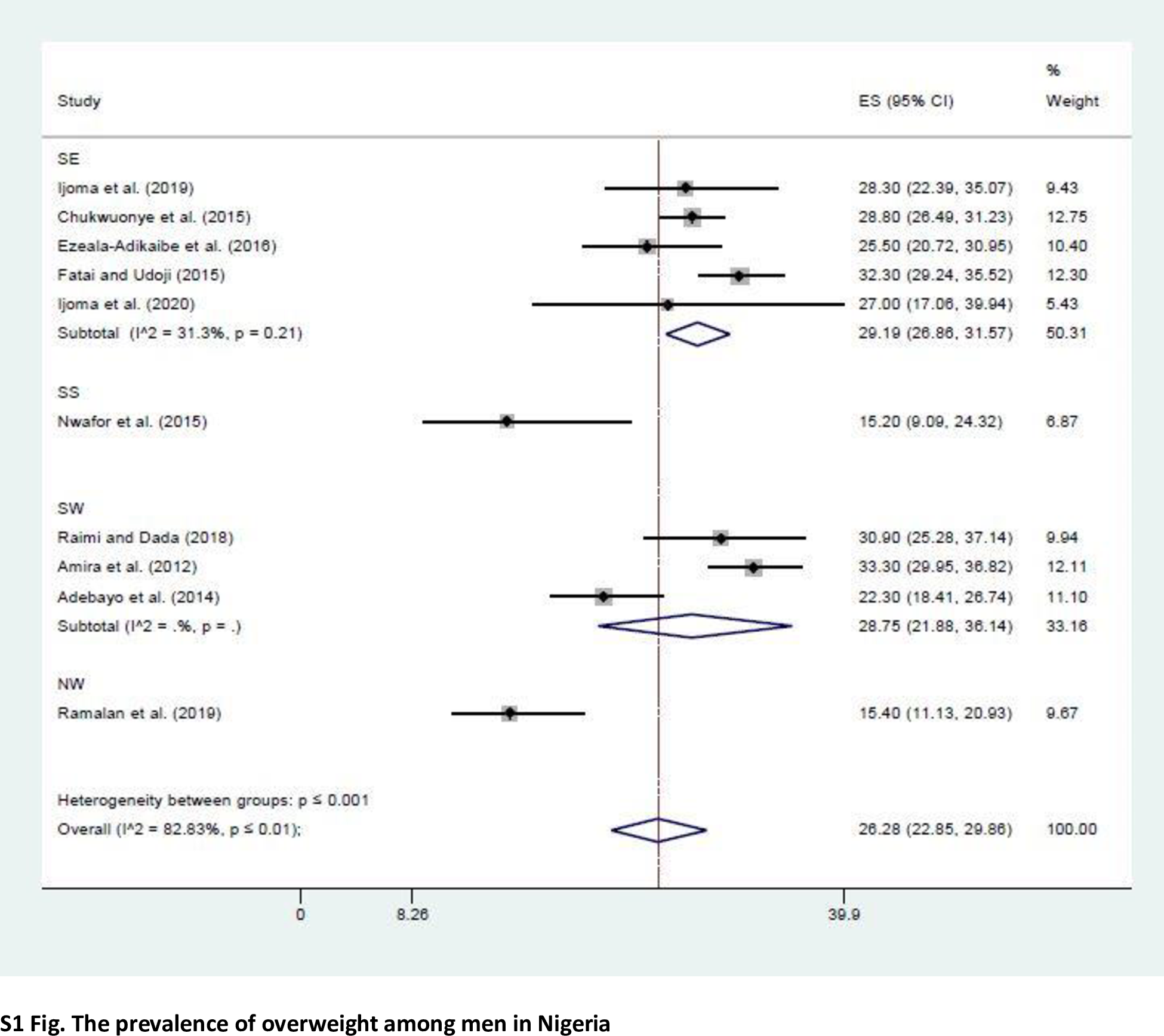

Supplement: S1 Fig — (TIF) [file pgph.0000515.s001.tif]

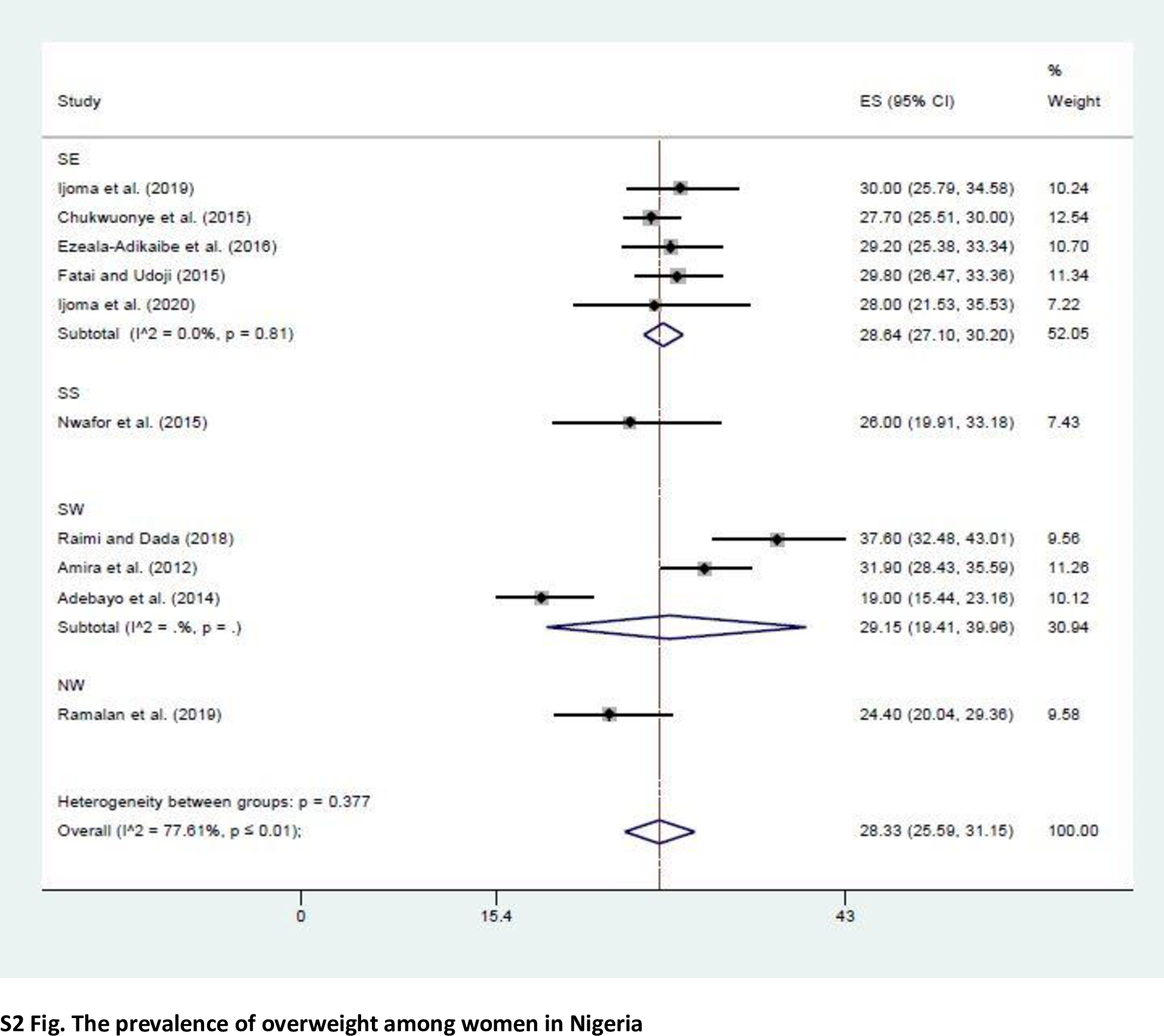

Supplement: S2 Fig — (TIF) [file pgph.0000515.s002.tif]

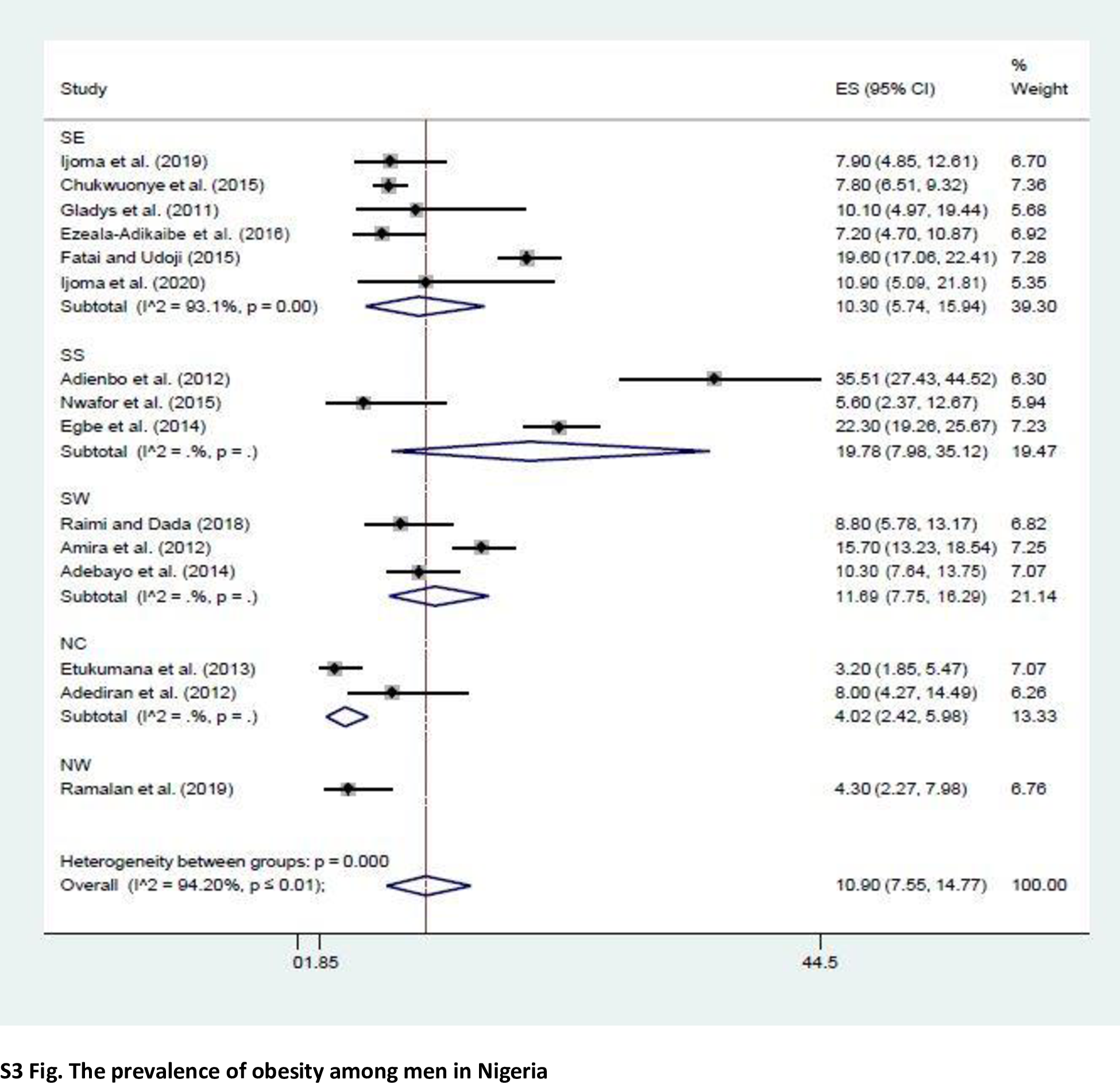

Supplement: S3 Fig — (TIF) [file pgph.0000515.s003.tif]

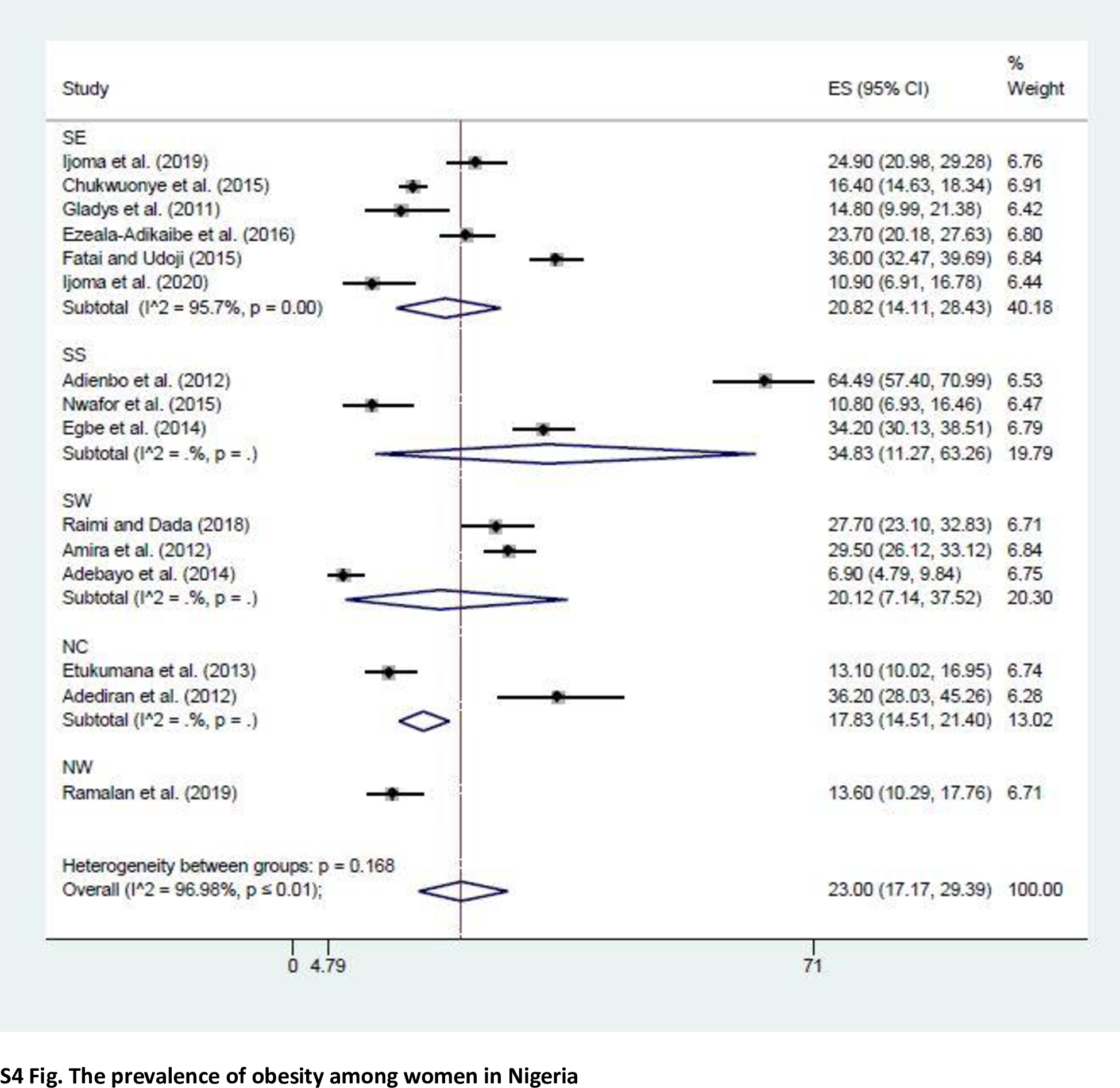

Supplement: S4 Fig — (TIF) [file pgph.0000515.s004.tif]
