# Supplementary material for: Prevalence of overweight and obesity in Nigeria: Systematic review and meta-analysis of population-based studies
Source: PLOS Glob Public Health. 2022 Jun 10;2(6):e0000515. doi: 10.1371/journal.pgph.0000515 (PMC10021772; doi:10.1371/journal.pgph.0000515)
Supplement: S1 Table — (DOCX) [file pgph.0000515.s005.docx]

**S1 Table. The prevalence of overweight among men and women in population based studies in Nigeria.**

| **Ref**  **Number** | **Author**  **(Year)** | **Total**  **Sample**  **Size** | **Men**  **Sample**  **size** | **Women**  **Sample**  **Size** | **Men**  **Prevalence**  **%** | **Women prevalence**  **%** |
| --- | --- | --- | --- | --- | --- | --- |
| 9 | Ijoma et al. (2019) | 605 | 191 | 414 | 28.3 | 30.0 |
| 10 | Chukwuonye et al. (2015) | 2928 | 1399 | 1529 | 28.8 | 27.7 |
| 20 | Ezeala-Adikaibe et al. (2016) | 774 | 276 | 498 | 25.5, | 29.2 |
| 21 | Fatai and Udoji (2015) | 1521 | 846 | 675 | 32.3 | 29.8 |
| 22 | Ijoma et al. (2020) | 210 | 55 | 155 | 27.0 | 28.0 |
| 26 | Nwafor et al. (2015) | 250 | 85 | 165 | 15.2 | 26.0 |
| 31 | Raimi and Dada (2018) | 552 | 230 | 322 | 30.9 | 37.6 |
| 35 | Amira et al. (2012) | 1368 | 720 | 648 | 33.3 | 31.9 |
| 37 | Adebayo et al. (2014) | 777 | 382 | 395 | 22.3 | 19.0 |
| 44 | Wahab et al. (2011) | 300 | Not stated | Not stated | 41.96 | 62.0 |
| 47 | Ramalan et al. (2019) | 532 | 208 | 324 | 15.4, | 24.4 |

^Wahab et al did not state men and women sample sizes and was removed from the further analysis
