# Supplementary material for: Prevalence of overweight and obesity in Nigeria: Systematic review and meta-analysis of population-based studies
Source: PLOS Glob Public Health. 2022 Jun 10;2(6):e0000515. doi: 10.1371/journal.pgph.0000515 (PMC10021772; doi:10.1371/journal.pgph.0000515)
Supplement: S2 Table — (DOCX) [file pgph.0000515.s006.docx]

**S2 Table. The prevalenceof obesity among men and women in population based studies in Nigeria**

| **Ref**  **Number** | **Author**  **(Year)** | **Total**  **Sample**  **size** | **Men**  **Sample**  **Size** | **Women**  **Sample**  **Size** | **Men**  **Prevalence**  **%** | **Women prevalence**  **%** |
| --- | --- | --- | --- | --- | --- | --- |
| 9 | Ijoma et al. (2019) | 605 | 191 | 414 | 7.9 | 24.9 |
| 10 | Chukwuonye et al. (2015) | 2928 | 1399 | 1529 | 7.8 | 16.4 |
| 19 | Gladys et al. (2011) | 218 | 69 | 149 | 10.1 | 14.8 |
| 20 | Ezeala-Adikaibe et al. (2016) | 774 | 276 | 498 | 7.2 | 23.7 |
| 21 | Fatai and Udoji (2015) | 1521 | 846 | 675 | 19.6 | 36 |
| 22 | Ijoma et al. (2020) | 210 | 55 | 155 | 10.9 | 10.9 |
| 12 | Adienbo et al. (2012) | 304 | 117 | 187 | 35.51 | 64.49 |
| 26 | Nwafor et al. (2015) | 250 | 85 | 165 | 5.6 | 10.8 |
| 27 | Egbe et al. (2014) | 1134 | 645 | 489 | 22.3 | 34.2 |
| 31 | Raimi and Dada (2018) | 552 | 230 | 322 | 8.8 | 27.7 |
| 35 | Amira et al. (2012) | 1368 | 720 | 648 | 15.7 | 29.5 |
| 37 | Adebayo et al. (2014) | 777 | 382 | 395 | 10.3 | 6.9 |
| 39 | Etukumana et al. (2013) | 750 | 385 | 365 | 3.2 | 13.1 |
| 41 | Adediran et al. (2012) | 229 | 113 | 116 | 8 | 36.2 |
| 44 | Wahab et al. (2011) | 300 | Not stated | Not stated | 9.3 | 29.8 |
| 47 | Ramalan et al. (2019) | 532 | 208 | 324 | 4.3 | 13.6 |

Wahab et al was excluded from further analysis the sample sizes for men and women not given in the article.
